# Transfer Learning Approach for Classification of Histopathology Whole Slide Images

**DOI:** 10.3390/s21165361

**Published:** 2021-08-09

**Authors:** Shakil Ahmed, Asadullah Shaikh, Hani Alshahrani, Abdullah Alghamdi, Mesfer Alrizq, Junaid Baber, Maheen Bakhtyar

**Affiliations:** 1 Department of Computer Science and Information Technology, University of Balochistan, Quetta 87300, Pakistan; shakil.mscs@um.uob.edu.pk (S.A.); junaidbaber@ieee.org (J.B.); maheenbakhtyar@um.uob.edu.pk (M.B.); 2 College of Computer Science and Information Systems, Najran University, Najran 61441, Saudi Arabia; hmalshahrani@nu.edu.sa (H.A.); aaalghamdi@nu.edu.sa (A.A.); msalrizq@nu.edu.sa (M.A.)

**Keywords:** deep learning, transfer learning, histopathology

## Abstract

The classification of whole slide images (WSIs) provides physicians with an accurate analysis of diseases and also helps them to treat patients effectively. The classification can be linked to further detailed analysis and diagnosis. Deep learning (DL) has made significant advances in the medical industry, including the use of magnetic resonance imaging (MRI) scans, computerized tomography (CT) scans, and electrocardiograms (ECGs) to detect life-threatening diseases, including heart disease, cancer, and brain tumors. However, more advancement in the field of pathology is needed, but the main hurdle causing the slow progress is the shortage of large-labeled datasets of histopathology images to train the models. The Kimia Path24 dataset was particularly created for the classification and retrieval of histopathology images. It contains 23,916 histopathology patches with 24 tissue texture classes. A transfer learning-based framework is proposed and evaluated on two famous DL models, Inception-V3 and VGG-16. To improve the productivity of Inception-V3 and VGG-16, we used their pre-trained weights and concatenated these with an image vector, which is used as input for the training of the same architecture. Experiments show that the proposed innovation improves the accuracy of both famous models. The patch-to-scan accuracy of VGG-16 is improved from 0.65 to 0.77, and for the Inception-V3, it is improved from 0.74 to 0.79.

## 1. Introduction

In the field of medical science, automatic analysis of histological images has created great convenience for doctors and scientists. Experts from different fields of computing and machine learning are able to contribute to medical science due to the availability of labeled data and technology that can digitize the data used in everyday analysis. Recently, in the field of pathology, it has become technologically easy to digitally scan the sample on the slides that are used for microscopy analysis and use it for computer-aided analysis and diagnosis. The digital scan of the sample on the slide is called a whole slide image (WSI) that enables the storage of the sample digitally on the computer in the shape of a digital image. The WSI can be used for detailed analysis and diagnosis by experts remotely or as a reference for future predictions. The saved WSI can easily be shared with experts in entirely different corners of the world for their swift analysis of the image. WSI processing has provided huge convenience for practitioners and has also motivated scientists to make more robust and reliable automatic analysis diagnostic models. Medical image analysis software is powered by machine learning, particularly, deep learning-based models. Deep learning with a convolutional neural network (CNN) is a quickly expanding field in histological image analysis. In a variety of image analysis fields, machine learning using a CNN has recently drawn the research community’s interest [1,2]. It provides physicians with an accurate analysis of diseases and helps them to correlate with previously stored samples, which leads to more effective medical decisions. For computerized applications, preliminary CNN-based architectures are proposed: including the use of magnetic resonance imaging (MRI) scans, computerized tomography scans, and electrocardiograms (ECGs) to detect life-threatening diseases, including heart disease, cancer, and brain tumors. Training deep learning models from scratch creates problems because state-of-the-art CNN requires a significant training size [3] and computational resources, as the WSI samples are comprised of gigapixels information [2]. CNNs, such as DenseNet [4], when trained on ImageNet, obtained high accuracy [5] because ImageNet has a huge databank of images for the training of CNN models. Deep features and transfer learning have allowed these deep models to be used in a variety of domains, including medical applications [5,6]. The methods for extracting features from histopathological images based on similarities between the feature vectors can be difficult when extracting data from a large database. Therefore, more advancement in the field of digital pathology is expected and needed. Microscopic analysis of histopathology images is time-consuming and difficult. Automated histopathology image diagnosis reduces pathologists’ workload and helps them to concentrate on more sensitive cases. 

Deep neural network (DNN) architecture is a versatile technique that has learned to perform complex tasks such as classification and facial recognition using a wide collection of images (ImageNet). Using “pre-trained” networks in medical image classification is a realistic way to use them. This solves the problem of not having a massive, well-labeled, and well-balanced image dataset. Babaie et al. have introduced the Kimia Path24 dataset. They applied LBP, the bag of visual word model, and two famous deep learning models. The highest accuracy based on their experiments was 41.80% from CNN models [7].

Deep models have performed well in several domains including medical applications and deep characteristics in medical images. There are alternatives for transfer learning, given domain data and a network that has been trained to differentiate on large nonspecific datasets (e.g., ImageNet, which has a huge databank of objects with more than 10,000 categories), the classification model must be adapted to the current domain using one of these methods: (a) The architecture is conditioned for several epochs after being initialized with random weights. The model learns characteristics from the data and computes weights using backpropagation at every single epoch. If the dataset isn’t large enough, this method will not be able to produce the most accurate results. It should be used as a reference point for the other two methods. (b) This approach uses weights trained on a wider dataset to initialize the model. A pre-trained CNN can be used as a feature extractor by freezing all convolutional blocks and then training the connected layers with the new dataset; it assumes that the layer just before the classifier is a feature layer instead of using the classifier of the pre-trained CNN; the classifier-like support vector machine and neural network can be used for the classification purpose. (c) This approach involves fine-tuning a pre-trained CNN as a classifier by retaining only the pre-trained network’s final layers (the domain layers) or by training models from scratch, in addition to retraining the classifier at the end of the fully connected network. The following are the major advantages of this research work:

i. All of the images in the Kimia Path24 database were used for training and testing purposes and were further classified into 24 classes for grayscale histopathology images.
ii. Training the entire VGG16 and Inception-V3 [8,9] models from scratch after transferring the pre-trained weights of the same model has improved classification accuracy as compared to fine-tuning (by training the last few layers of the base network) or using high level feature extractor techniques for the classification of grayscale images in the Path24 dataset.
iii. The proposed pre-trained CNN models have fully automated the end-to-end structure and do not need any hand-made feature extraction methods.

In this paper, we analyze and assess the effectiveness of Inception-V3 and VGG-16 pre-trained models for Kimia Path24 using the transfer learning approach, which involves full training of the pre-trained models by fine-tuning early layers for the automatic classification of histopathology images. Following is a review of the paper’s structure: Section 2 presents a concise overview of the applicable literature. Section 3 explains the Dataset and Methodology in detail. The Experiment, Results, and Discussion are given in Section 4. Section 5 covers the conclusion.

## 2. Related Works

Pre-trained models, which have been trained on a huge databank of images, are used as feature extractors or weight initializers for the classification of histopathological images [6,7,8,9,10]. The high dimensionality of digital pathology images makes processing and storage difficult [2]; therefore, using soft-computing approaches and understanding regions of importance in an image helps in quicker diagnosis and identification [11]. Scanning and segmentation, as well as detection and retrieval, are all traditional image processing tasks that have increased in importance over time. It can be seen that cell structures such as nuclei, glands, and lymphocytes have outstanding features, which can be used as indicators to identify cancer cells, especially in histopathology [12]. However, digitization of whole slide images is setting a landmark for laboratory standards, for more accurate and speedy diagnoses of diseases [13].

Image extraction and image classification are the main components of pathological images in histopathology whole slide image (WSI) analysis [14,15]. These help medical doctors to make more specific and accurate decisions on the patient’s medical condition. There are several benefits of digitizing pathology images. Additionally, the better presentation of image processing algorithms can make the retrieval of images more efficient. Clinicians and quality management staff can take advantage of this property. The digitized version of pathology glass slides is one of the most recent and prominent examples of extensive automated evidence [16]. The size of whole scan images of pathology samples can be in gigabytes [2,17]. As a result, storing, processing, and transferring images in real-time is complicated. Yet, learning deep features from massive, digitized histopathology scans is a decent way to discover secret patterns that humans cannot recognize. Furthermore, pathology image processing is now considered the “gold standard” of diagnosing multiple diseases involving all forms of cancer [18].

Over the past few years, clinicians and researchers have been interested in machine learning techniques for the automated analysis of digital pathology scans. With advantages of high variety, rich structures, and wide dimensionality, these images come with special challenges. As a result, scholars have been looking at different image processing methods and how they can be applied to digital pathology [13]. The use of deep features as image descriptors is a fairly new advancement, mainly based on CNNs, which are trained from initial layers or use post-training for classification to extract high-dimensional characteristics embedded in the fully connected layer [19,20,21]. CNNs and several other discriminative deep architectures need optimal training on a large amount of labeled (and balanced) data without the adverse effects of overfitting [22,23,24]. In histopathology image extraction, deep solutions have been widely used. In [25], to extract features from histopathology files, a sparse autoencoder was used. The authors of [26] demonstrated a patch-based CNN and proposed an expectation–maximization (EM) technique for training CNN. The author of [27] purposed a CNN-based nuclei-guided feature extraction technique for histopathological imaging. In addition, there are a number of frameworks based on handcrafted features [28,29,30,31,32]. 

The use of pre-trained networks for operations outside of their original domain has gained attention [33]. This is especially relevant in the medical field, and the most obvious reason for this is the lack of sufficient labeled data that is needed by a deep network for training purposes. When it comes to using pre-trained networks for medical imaging studies, these groups have achieved better results [33,34,35]. Hence, other organizations have used ImageNet (a huge databank of images, divided into 1000+ categories) for the training of networks [35,36]. Kieffer et al. used Kimia Path24 to look into the use of deep features by using pre-trained architectures, adjusting for the effects of transfer learning, and comparing pre-trained networks against training from scratch [37]. 

Later in this section, some famous and recent works on deep learning-based models for medical imaging are discussed.

In recent years, CNN has improved the accuracy of medical image classification tasks from traditional diagnosis to automatic diagnosis, reaching different levels with excellent performance. An example of these tasks is the diagnosis of breast cancer. Hematoxylin and eosin-stained breast biopsy images fall into four categories: invasive carcinoma, in situ carcinoma, benign tumor, and normal tissue. Saha et al. [38] proposed an automatic disease detection of mitoses from breast histopathology WSIs with precision of 0.92 and recall of 0.88. Han et al. [39] proposed a framework to distinguish breast cancer histopathology photos using a hierarchical deep learning model. Their purposed system divided the subcategories of breast cancer imaging into three categories (lobular carcinoma, ductal carcinoma, and fibroadenoma) with an overall accuracy of 0.93. Zheng et al. [27] created a CNN to categorize breast cancer photographs into two groups (benign and malignant) with precision of 0.96 on their dataset. Jia et al. [40] used a multi-instance learning algorithm to implement a fully connected network to segment cancer areas on histopathological images. Xu et al. [41] used the transfer learning approach; CNN was applied to segment and label histopathology WSIs. Shi et al. [42] applied a deep hashing method to retrieve and classify the histopathology images. The suggested model was tested on a dataset of lung cancer by scientists, and the model reported accuracy of 0.97. Another study [43] suggested three different CNN models to classify the coronavirus contamination in X-radiation cases, including Inception-ResNetV2, InceptionV3, and ResNet50. In terms of detection and identification, the ResNet50 system outperformed InceptionV3 and Inception-ResNetV2 with 0.98 accuracy, whereas InceptionV3 attained 0.97, and Inception-ResNetV2 attained 0.87. An ensemble-based framework to classify in vivo endoscopic images as normal or abnormal using VGG, DenseNet, and inception-based networks was proposed [44]. Sari et al. [45] proposed a semi-supervised classification scheme based on a restricted Boltzmann machines to classify histopathological tissue images. They regulate the noticeable subregions of an image and quantify the image by employing the chrematistics of these subregions but without considering the image locations as a whole. Wang et al. [46] proposed a weakly-supervised learning-based framework for classification of WSIs of lung cancer. They used a fully convolutional network to generate the potential regions that are likely to be the cancer regions. They also demonstrated that CNN-based features are more robust and discriminative compared to the handcrafted features. 

Pathologists examine pathology slides at various resolutions and fields of view in a similar manner. Nonetheless, like many others, we use a deep learning approach on minor portions of the image. By doing this, the classifier is expanded to each element of the entire slide. This study used WSIs from the Kimia Path24 dataset, which is specially designed to examine the classification and retrieval of histopathology images. In total, there were 1325 images for the test and 22,590 for training because the DNN work on raw pixel values requires no extra efforts from humans and can learn a variety of graphical characteristics from the data held for training.

## 3. Material and Methods

Transfer learning is widely used for various applications. Pre-trained models learn small patterns such as shapes and diagonals in the initial layer and then combine these components in subsequent layers to learn multipart features. By using patterns learned from previous layers, the models make meaningful constructs in the final layer. 

### 3.1. Proposed Model

We take two famous models for feature extraction and then use those features to train the models. The two models are VGG16 [8] and Inception-V3 [9]. The VGG16 was proposed for ImageNet competition in 2014. The main appealing factor of this model is the use of a filter size of 3 × 3 with stride 1 rather than having a very large number of hyperparameters. The last layer before the concatenation layer contains the feature of length 4096. The Inception network was proposed by Google in 2014 with 22 layers comprised of 5 million parameters with different filter sizes of 1 × 1, 3 × 3, and 5 × 5. These filters were used with different scales to extract the features. Later, in 2015, Google proposed Inception-V3 with reduced parameters without hurting the accuracy of the model [47]. Both models, VGG-16 and Inception-V3, are widely used for various applications.

**Figure 1:**
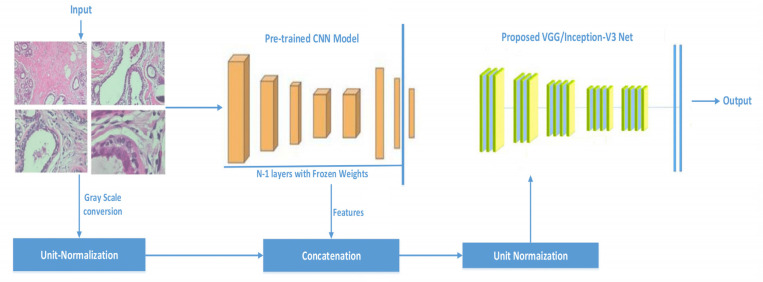
Proposed framework based on transform learning; the given image is passed to the pre-trained network and features are extracted, later, that feature is concatenated with the same image which is vectorized by unit normalization.

The difference between existing practices and the proposed methodology is that we concatenate the features extracted from existing models with the processed images and then train the model from scratch, as shown in Figure 1. It can be seen that weights from previously trained models are transferred to the same architecture by infusing the weights with raw image pixel values. To project weights and the pixel values in the same feature space, unit normalization before concatenation and after concatenation is performed. Feature concatenation during training is widely used [48]. However, we concatenate pre-trained weights with image raw pixel values. We trained all of the network’s layers because of their ability to extract both common and individual functions. By doing so, we are able to pass to the new model information (weights values) about simple features gained in the first and middle layers. Histopathology images are classified using the basic constructs that purposed CNN models have learned in order to distinguish various images from the ImageNet. The following are the major contributions of this work:

- Inception-V3 and VGG16 are evaluated for classifying histopathology images automatically.
- The classification effectiveness of purposed pre-trained models is tested by infusing the features vectors from pre-trained network with image pixels normalized. We used grayscale histopathology images.

By training Inception-V3 and VGG16 models by transferring the weights of the same models that are trained on very large and independent datasets, the accuracy of classification of histopathology images was increased. Fine-tuning and feature extractor-based experiments have already been conducted by many recent papers. However, we take the features from pre-trained models and concatenate them with an original image before training the model form scratch. Our framework is inspired by the feature concatenation approach of [48].

### 3.2. Dataset

**Figure 2:**
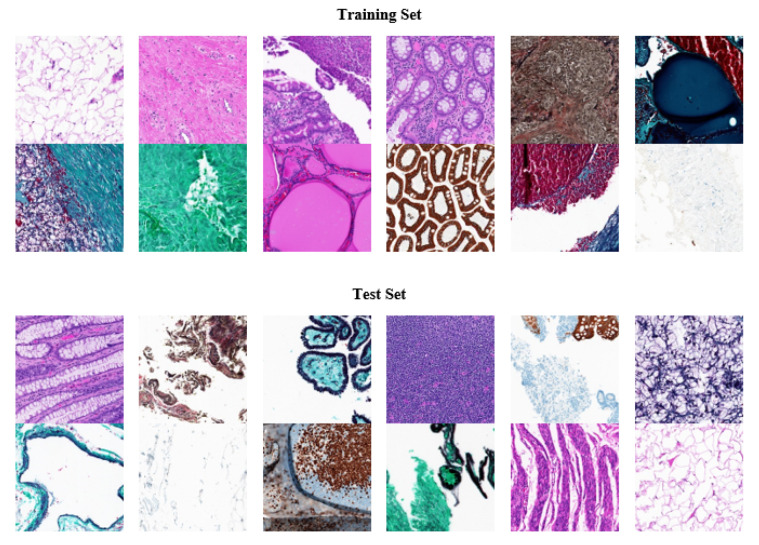
Random sample images from training set and test of Kimia Path24 dataset.

We used Kimia Path24, an open-source dataset with histopathology images, to analyze our tests. It was designed with digital pathology image classification and retrieval in mind. The dataset was created using 350 whole scan images (WSIs) of different body parts. Different staining techniques were applied such as immunohistochemical (IHC), hematoxylin and eosin (H&E), and Masson’s trichrome staining. Tissue-Scope LE 1.0 was used to record the images in the bright field with a 0.75 NA lens http://www.hurondigitalpathology.com/tissuescope-le-3/ (accessed on 8 August 2021). A total of 24 WSIs were chosen for nonmedical experts based on visual differentiation. There were 22,591 training instances and 1325 testing instances provided each of resolution of 1000 × 1000 pixels (0.5mm × 0.5mm) from 24 classes [49]. The dataset is quite challenging and computationally expensive due to high dimensions of the images. Figure 2 shows some colored images from the dataset; the dataset is freely available online https://kimialab.uwaterloo.ca/kimia/index.php/pathology-images-kimia-path24/ (accessed on 8 August 2021).

### 3.3. Accuracy Calculation

The final accuracy calculation for the Kimia Path24 dataset is based on two types of accuracy calculation, namely path-to-scan and whole-scan accuracies, established by [7]. 

The total number of test patches is denoted by $n_{tot}$ and for the dataset $n_{tot}$ = 1325. There are 24 different classes (one for each whole slide image) denoted by set *S*, i.e., *S* = {c_0_, c_1_, …, c_23_}. Any given test patch from the dataset is denoted by $P_{s}^{i}$, where *s* ∈ *S* represents its class and *i* ∈ [1, $n_{\Gamma_{s}}$] is index to identify it among all the patches associated with class *s*. The $\Gamma_{s}$ is set of patches $P_{s}^{i}$ that belongs to class *s* such that $\Gamma_{s}$ = $\left\{P_{s}^{i}|s\in S,i=1,2\ldots ,n_{\Gamma s}\right\}$ with $n_{\Gamma_{s}}$ is number of patches in $s^{th}$ class.

Patch-to-scan accuracy $\eta_{p}$ is calculated using Equation (1), where R represents the retrieved images for each experiment

(1) $$\eta_{p}=\frac{\sum_{s\in S}\left|R\cap \Gamma_{s}\right|}{n_{tot}}$$

and the whole-scan precision $\eta_{w}$, which is expressed as Equation (2).

(2) $$\eta_{w}=\frac{1}{24}\underset{s\in S}{\sum }\frac{|R\cap^{\;}\Gamma_{s}|}{\eta_{\Gamma_{s}}}$$

Overall precision is calculated, as shown in the Equation (3) [7].

(3) $$\eta_{total}=\eta_{p}.\eta_{w}$$

## 4. Experiments and Results

VGG-16 and Inception-V3 were employed to categorize Kimia Path24 grayscale histopathology into 24 categories. Later in this section, the experimental setup is explained supported by the results and discussion.

### 4.1. Experimental Setup

As stated earlier, transfer learning was used to train the models, i.e., VGG16 and Inception-V3. The pre-trained models were trained on very large datasets; we provided the image as input to that layer and extracted the features from the n-1 layer, then, that feature was concatenated with the unit normalized image. To normalize the values in the concatenated vector, the whole vector was unit normalized again, as demonstrated in Figure 1. 

During training, Adamax was used to refine the network parameters. The learning rate of $10^{-6}$ was selected. To regularize the deep models, a dropout ratio of 0.25 was chosen. All hyperparameters were selected based on experimental trials. Initial values were taken as suggested by their original papers. In the case of VGG-16, the suggested value of dropout ratio is 0.5, which was not optimal in our validation trial. The different batch sizes were trialed during learning, the batch sizes were chosen from 30 to 150 due to hardware constraints, and the optimal batch size we obtained was 140. The larger batch size can also be taken if the training size is increased, either by expert annotation or data augmentation.

Moreover, the Inception-V3 and VGG16 original models with their default configuration were also trained from scratch on the same dataset. Python, version 3.7.11, with Keras Chollet, F. “Keras”, https://github.com/fchollet/keras, 2015 (accessed on 8 August 2021), version 2.5.0, were used on Google Colab. Kimia Path24 contains a total of 23,915 images; the dataset is divided by the publishers into two sets, a training set that contains 22,590 images, and a test set that contains 1325 images. The test set is 5.5% of the whole dataset. To make training more robust, the training dataset is further divided into two sets: training that is 80% of the 22,590 images and validation which is 20% of the 22,590 images. The scripts and models can be accessed online https://github.com/shakil1987/transfer_learning_on_WSI (accessed on 8 August 2021).

### 4.2. Results

**Figure 3:**
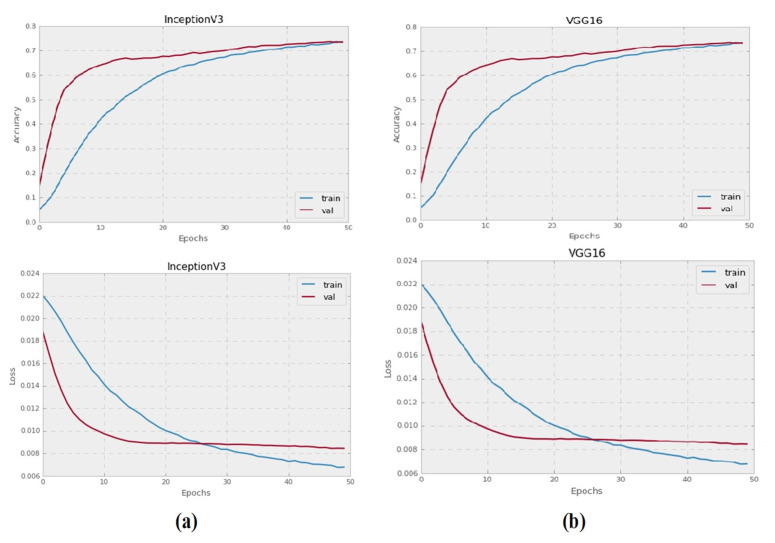
The performance graphs of Inception-V3 and VG16 pre-trained models on grayscale images: (**a**) validation accuracies, and training and validation loss of Inception-V3 model on grayscale images, (**b**) validation accuracies, and training and validation loss of VGG16 on grayscale images.

The Inception-V3 and VGG16 pre-trained CNN models were trained to categorize grayscale histopathology images for 50 epochs. All images with a resolution of 128 × 128 pixels were used to test each pre-trained model. Figure 3 illustrates the training and validation failure curves for VGG16 and Inception-V3, as well as the validation precision. The experiments show that there was no accuracy gain after 50 epochs, instead, accuracy started to deteriorate. 

**Figure 4:**
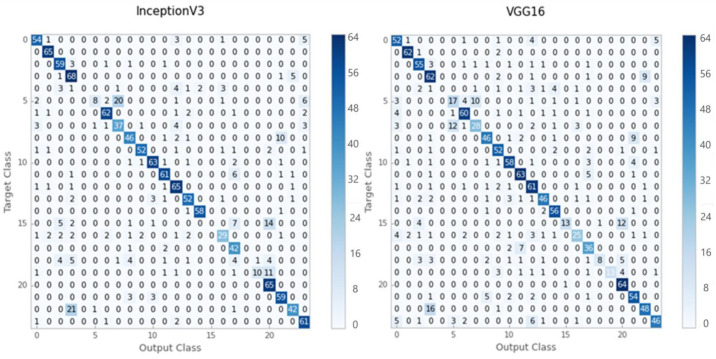
The confusion matrixes for the Inception-V3 and VGG16 pre-trained models.

According to the results of the evaluation, Inception-V3 provided better classification accuracy for grayscale images than VGG-16. Figure 4 illustrates the confusion matrices obtained using the Inception-V3 and VGG16 models.

In the grayscale test dataset, the Inception-V3 model correctly classified 1058 out of 1325 images, while the VGG16 model correctly classified just 1025 out of 1325 images. Table 1 shows the accuracy, recall, and F1-score values of the Inception-V3 and VGG16 models for grayscale test-set images. Inception-V3 yielded 80 percent for the average precision, recall, and F1 score for grayscale histopathology images. On the other hand, VGG16 achieved 77 percent using the same assessment criterion.

It can be seen that a few of the classes have zero precision and, among them, there are a small number of instances for the training and testing sets. 

### 4.3. Discussion

Kieffer et al. [37], to categorize histopathology WSIs in Kimia Path24, used Inception-V3 and VGG-16 models with fine-tuning and feature extraction approaches. On the same dataset (Kimia Path24), Table 2 provides a comparison of our work with state-of-the-art frameworks.

To categorize grayscale histopathology images, Babaie et al. suggested a bag of words (BoW), local binary pattern (LBP) histograms, and a CNN model in 2017. For the BoW, LBP, and CNN methods, the absolute accuracy value (𝜼 𝒕𝒐𝒕𝒂𝒍) was recorded as 39.65 percent, 41.33 percent, and 42.07 percent, respectively.

Kieffer et al. [37] employed Inception-V3 and VGG16 pre-trained CNN models to categorize histopathology images using transfer learning techniques. For grayscale histopathology images, Inception-V3 with 74.87 percent obtained maximum precision.

It can be seen from Table 2 that the Inception-V3 and VGG16 accuracy improved after concatenating their pre-trained weights with image pixel values. Before concatenation, the pre-trained feature vector and image are unit normalized so that they are on the same Euclidean space, even after concatenation, and the final vector is unit normalized. Transferring of the pre-trained weights to the same model for training actually improves the accuracy of the same model. The results for total accuracy on the test set were 57.00% for the Inception-V3 and 55.17% for VGG16. The accuracy of the original VGG-16 and Inception-V3 is also competitive without transfer learning. The main reason that VGG-16 and Inception-V3 gave better performance compared to the work of Babaie et al. is the fact that both of these models are deep and also the training dataset was comparatively small for these two. The proposed model, on the other hand, uses the same two models but achieves a better performance, and also is comparatively more generalized as, besides the dataset used in the experiments, the weights from these two models, which are trained on millions of images, are also incorporated during the training. 

## 5. Conclusions

The adoption of deep learning in digital pathology would be extremely beneficial as it would move human appraisal of histology to higher quality, nonrepetitive takes. Deep learning provides pathologists with the ability to analyze data at high speeds while maintaining accuracy. For the automatic classification of histopathology images, this paper proposes training the entire pre-trained model from pre-trained weights concatenated with image raw pixels. According to the findings, the pre-trained models, Inception-V3 and VGG-16, outperformed existing studies in the literature for Kimia Path24 grayscale histopathology scans. Both models had better patch-to-scan accuracy: VGG-16 had a noticeable increase in total accuracy whereas Inception-V3 had a slight improvement. 

The main limitations of the study are the size of the concatenated vector and the size of the dataset used for the training of pre-trained models. We may have obtained better accuracy if we had been able to access to a larger number of samples, such as millions of histopathology WSIs, and also if the purposed models were trained in medical imaging because the architecture was adjusted appropriately for research work. The training dataset is also imbalanced as some of the classes had only a few examples. In future work, we are interested in exploring deep models for data augmentation to address the imbalanced nature of the dataset. 

## Figures and Tables

**Table 1:** The grayscale research dataset was used to create a classification report for the Inception-V3 model.

Classes	Amount of Data	Precision		Recall		F1-Score	
		Inception-V3	VGG16	Inception-V3	VGG16	Inception-V3	VGG16
c0	64	0.83	0.70	0.84	0.81	0.84	0.75
c1	65	0.92	0.94	1.00	0.95	0.96	0.95
c2	65	0.80	0.80	0.91	0.85	0.85	0.82
c3	75	0.64	0.66	0.91	0.83	0.75	0.73
c4	15	0.00	0.00	0.00	0.00	0.00	0.00
c5	40	0.80	0.52	0.20	0.42	0.32	0.47
c6	70	0.90	0.83	0.89	0.86	0.89	0.85
c7	50	0.63	0.70	0.74	0.56	0.68	0.62
c8	60	0.79	0.79	0.77	0.77	0.78	0.78
c9	60	0.93	0.76	0.87	0.87	0.90	0.81
c10	70	0.90	0.83	0.90	0.83	0.90	0.83
c11	70	0.87	0.82	0.87	0.90	0.87	0.86
c12	70	0.76	0.70	0.93	0.87	0.84	0.78
c13	60	0.85	0.84	0.87	0.77	0.86	0.80
c14	60	0.97	0.84	0.97	0.93	0.97	0.88
c15	30	0.00	0.93	0.00	0.43	0.00	0.59
c16	45	0.81	0.76	0.64	0.56	0.72	0.64
c17	45	0.65	0.68	0.93	0.80	0.76	0.73
c18	25	0.00	0.89	0.00	0.32	0.00	0.47
c19	25	0.91	1.00	0.40	0.52	0.56	0.68
c20	65	0.68	0.74	1.00	0.98	0.81	0.85
c21	65	0.80	0.77	0.91	0.83	0.85	0.80
c22	65	0.84	0.80	0.65	0.74	0.73	0.77
c23	65	0.78	0.82	0.94	0.71	0.85	0.76

**Table 2:** Comparison of proposed innovation with famous state-of-the art models.

Paper	Model	Method	$\eta_{p}\;(\%)$	$\eta_{w}\;(\%)$	$\eta_{total}\;(\%)$
Babaie et al. [7]	CNN	Train from scratch	64.98	64.75	42.07
Kieffer et al. [37]	VGG-16	Feature Extractor	65.21	64.96	42.36
Kieffer et al. [37]	VGG-16	Fine-tuning	63.85	66.23	42.29
Kieffer et al. [37]	Inception-V3	Feature Extractor	70.94	72.24	50.54
Kieffer et al. [37]	Inception-v3	Fine-tuning	74.87	76.10	56.98
Simonyan et al. [8]	VGG-16 base model	Train from scratch	69.89	71.09	49.68
Szegedy et al. [9]	Inception-V3 base model	Train from scratch	72.65	73.00	53.03
Proposed model	VGG-16	Feature Extractor	77.41	71.27	55.17
Proposed model	Inception-V3	Feature Extractor	79.90	71.33	57.00

## Data Availability

All the data is available within the article.
